# Neural Correlates of Post-Conventional Moral Reasoning: A Voxel-Based Morphometry Study

**DOI:** 10.1371/journal.pone.0122914

**Published:** 2015-06-03

**Authors:** Kristin Prehn, Marc Korczykowski, Hengyi Rao, Zhuo Fang, John A. Detre, Diana C. Robertson

**Affiliations:** 1 Cluster of Excellence “Languages of Emotion”, Freie Universität Berlin, Berlin, Germany; 2 Department of Neurology & NeuroCure Clinical Research Center, Charité Universitätsmedizin Berlin, Berlin, Germany; 3 Center for Functional Neuroimaging, Department of Neurology, University of Pennsylvania, Philadelphia, United States of America; 4 Department of Legal Studies & Business Ethics, The Wharton School, University of Pennsylvania, Philadelphia, United States of America; 5 Department of Psychology, Sun Yat-Sen University, Guangzhou, China; Penn State University, UNITED STATES

## Abstract

Going back to Kohlberg, moral development research affirms that people progress through different stages of moral reasoning as cognitive abilities mature. Individuals at a lower level of moral reasoning judge moral issues mainly based on self-interest (personal interests schema) or based on adherence to laws and rules (maintaining norms schema), whereas individuals at the post-conventional level judge moral issues based on deeper principles and shared ideals. However, the extent to which moral development is reflected in structural brain architecture remains unknown. To investigate this question, we used voxel-based morphometry and examined the brain structure in a sample of 67 Master of Business Administration (MBA) students. Subjects completed the Defining Issues Test (DIT-2) which measures moral development in terms of cognitive schema preference. Results demonstrate that subjects at the post-conventional level of moral reasoning were characterized by increased gray matter volume in the ventromedial prefrontal cortex and subgenual anterior cingulate cortex, compared with subjects at a lower level of moral reasoning. Our findings support an important role for both cognitive and emotional processes in moral reasoning and provide first evidence for individual differences in brain structure according to the stages of moral reasoning first proposed by Kohlberg decades ago.

## Introduction

Kohlberg’s cognitive-developmental approach dominated moral psychology for decades. Kohlberg’s theory assumes that individuals transition to higher levels of moral reasoning [1]. A key contributor to this stream of research is Rest, who devised the Defining Issues Test (DIT-2) to test which cognitive schema an individual uses when reasoning about moral issues [2]. Specifically, he proposed three cognitive schemas mapping Kohlberg’s three stages of moral reasoning: personal interests (pre-conventional level), maintaining norms and following conventional rules and laws (conventional level), and deeper principles or shared ideals (post-conventional level). A complementary approach to understanding individual differences in moral reasoning is offered by Lind [3]. Here, morality is defined as consisting of two inseparable, yet distinguishable aspects: moral values and preferences and a person’s competence to act accordingly. While the level of moral reasoning can be described in terms of moral values and preferences, moral judgment competence is the ability to apply these moral values in a consistent and differentiated manner in challenging social situations. Thus, moral judgment competence involves proficiency in application, whereas moral reasoning is concerned with the role of conscious and rational reasoning processes.

Neuroscience has recently reinvigorated moral psychology by introducing new methods for studying moral decision making [4]. Moral neuroscience has addressed three major issues: the role of reason and emotion in guiding moral decision making [5, 6], the unified vs. dual system organization for carrying out moral decision making in the brain, as well as content-specificity vs. domain-generality of moral decision making. This research has identified roles for specific brain regions supporting moral cognition including numerous sectors of the prefrontal cortex (PFC), limbic regions such as the amygdala, the temporal poles, the posterior cingulate cortex, and the posterior superior temporal sulcus (pSTS) [7, 8]. Research has also demonstrated age-dependent functional changes in neural circuits supporting moral cognition, specifically in the ventromedial prefrontal cortex (vmPFC) [9]. Higher moral judgment competence has been linked to reduced activity in the dorsolateral prefrontal cortex (dlPFC) and other regions of the moral cognition network (vmPFC/pSTS) [10]. Differences in moral values have also been linked with regional gray matter (GM) volume in a study by Lewis et al. [11]. Here, the values of harm/care and fairness were positively associated with dorsomedial PFC volume while authority, loyalty, and sanctity were positively associated with subcallosal gyrus volume. However, no study to date has quantified brain structures supporting individual differences in Kohlbergian stages of moral reasoning or moral judgment competence.

The present research adds an investigation of individual differences in moral reasoning to the expanding landscape of moral neuroscience. We used voxel-based morphometry (VBM) to investigate whether the stage of moral reasoning and moral judgment competence are reflected in structural brain architecture. According to literature, elaborated and controlled reasoning processes are represented by activity in the PFC [12]. Therefore, we expected that subjects with post-conventional schema preference and higher moral judgment competence would be characterized by increased GM volume in the PFC. Psychometric testing was also included to explore potential links between personality variables and individual differences in moral reasoning.

## Materials and Methods

The study was approved by the Institutional Review Board of the University of Pennsylvania. Written informed consent was obtained from each subject before the study. Subjects were compensated for their participation.

### Subjects

Our overall sample consists of 730 students enrolled in a two-year Master of Business Administration (MBA) program. Sample selection was intended to result in a relatively homogenous group with respect to age, educational level, and current educational experience. At the same time, the sample is heterogeneous in terms of undergraduate areas of study. Of the graduate students in the sample, 27% have undergraduate business majors, 23% majored in engineering, math, or the hard sciences, 45% in the humanities and social sciences, and 5% in other fields. The mean age for all study participants was 27.1 years with a range of 24 to 33 years. In this age group, structural brain maturation and, in particular, maturation of the frontal lobe is largely complete. All subjects had a similar educational background and multiple years of formal education, including a minimum four year undergraduate college degree. This is important, because both levels of moral reasoning and moral judgment competence are positively associated with number of years of formal education. In particular, it has been demonstrated that formal ethics training and educational programs that challenge people to look at issues from different points of view are effective in influencing people’s awareness of moral issues as well as moral judgment competence [3, 13].

As reported in the Introduction, there are two complementary approaches to understanding individual differences in moral reasoning. While the level of moral reasoning (or moral schema preference according to the theories of Kohlberg and Rest) is related to moral values and preferences, moral judgment competence (according to Lind’s theory) describes the ability to consistently apply these moral values and preferences in challenging social situations. In our study, we aimed to investigate the neural correlates of individual differences in the level of moral reasoning and moral judgment competence and included subjects in such a way that the two factors: level of moral reasoning and moral judgment competence were independently varied in a 2 x 2 factorial design.

As a first step, the entire sample completed the Moral Judgment Test (MJT) [14,15] on line. The MJT measures moral judgment competence and confronts a participant with two moral dilemmas as well as six arguments supporting (i.e., “pro-arguments”) and six arguments rejecting (i.e., “counter-arguments”) the protagonist’s solution. The participant is required to rate each argument with regard to its acceptability on a nine point rating scale ranging from -4 (highly unacceptable) to +4 (highly acceptable). Each argument represents a certain level of moral reasoning. The moral judgment competence score (C score) is calculated based on an individual’s total response variation in the quality of the given arguments. The C score reflects the degree to which a participant’s judgments about the pro- and counter-arguments are consistent. A high C score indicates that an individual consistently appreciates arguments referring to a specific moral reasoning stage. Conversely, low competence indicates that an individual inconsistently applies idiosyncratic arguments only supporting his or her own solution of the dilemma. In the absence of norms with regard to moral judgment competence, we defined subjects in this entire sample as “high” morally competent when having a high C score above the 85th percentile (C score = 10.93) and subjects as “low” morally competent when having a low C score below the 15th percentile (C score = 48.91).

As a second step, subjects with either “low” or “high” C scores were asked to complete an online version of the DIT-2. The DIT-2 measures the current level of moral reasoning an individual has reached during development. In this test, complex moral dilemmas (e.g., medically assisted suicide) are presented and participants are required to read each dilemma, as well as twelve rationales which provide arguments and responses to the dilemma. The participant is asked to evaluate and choose the relevance of each rationale. Participants’ responses were scored by the Center for the Study of Ethical Development at the University of Alabama, the publisher of the DIT-2. Based on schema preference, each participant was assigned to a specific type level: Type 1—consolidated personal interests schema, Type 2—transitional personal interests schema, Type 3—transitional maintaining norms schema (personal interests schema is secondary), Type 4—consolidated maintaining norms schema, Type 5—transitional maintaining norms schema (post-conventional schema is secondary), Type 6—transitional post-conventional schema, and Type 7—consolidated post-conventional schema. In addition to the schema type, the DIT-2 provides a metric score (among other scores) reflecting the extent to which a person prefers post-conventional moral thinking (N2 score).

This two step screening process using the MJT and the DIT-2 allowed us to select 67 subjects in the end for MRI scanning according to the following stratification:

1. Participants with post-conventional schema preference (post-con, Types 6 and 7) and high C score (above the 85th percentile; *n* = 21; 11 males),
2. Participants with post-conventional schema preference and low C score (test score below the 15th percentile; *n* = 17; 10 males),
3. Participants with personal interest and maintaining norms schemas (pre-con + con: Types 1 to 5) and high C score (*n* = 12; 6 males), and
4. Participants with personal interest and maintaining norms schemas and low C score (*n* = 17, 10 males).

The four groups did not differ with regard to age (F(3,66) = 1.292, p = .272) and gender distribution (chi2(1) = 0.731, p = 3.92). To control for differences in personality variables, all 67 subjects also completed the NEO Personality inventory (NEO PI-R) [16], the Rosenberg Self Esteem Scale [17], the Brief Locus of Control Scale [18], the General Self Efficacy Scale [19], and the Sense of Control Scale [20]. The NEO Personality Inventory, which is well recognized as a leading measure of personality assessment, is a 240 item questionnaire of global personality domains including Extraversion, Agreeableness, Conscientiousness, Neuroticism, and Openness to Experience. For technical reasons, NEO PI-R data from two subjects were not available.

### MRI data acquisition and quantitative voxel-based morphometry

For MRI data acquisition, we used a 3.0 T MRI scanner (Siemens Trio, Erlangen, Germany) with an 8-channel head receiver array. The structural scan consisted of 176 slices and was acquired in sagittal plane using a high-resolution T1-weighted magnetization-prepared rapid acquisition with gradient echo (MPRAGE) sequence (repetition time = 12.24 ms, echo time = 3.56 ms, flip angle = 23°, matrix = 256 x 256, voxel size: 1 x 1 x 1 mm).

Image analysis was performed using the VBM toolbox (http://dbm.neuro.uni-jena.de/vbm/) in SPM8 (Wellcome Department of Cognitive Neurology, London; http://www.fil.ion.ucl.ac.uk/spm) implemented in MATLAB 7.11.1 (Mathworks Inc., Sherborn, MA, USA). Data preprocessing consisted of tissue classification and segmentation into gray and white matter, image registration, as well as bias correction for magnetic field inhomogeneities. Additionally, Hidden Markov Random Fields (HMRF) were applied to increase the signal-to-noise ratio of the final tissue maps. HMRF provide spatial constraints on tissue segmentation based on the intensities of neighboring voxels. Specifically voxels which are isolated and unlikely to be associated with a certain tissue class are removed from the final tissue maps [21]. All resulting gray and white matter images were registered to a template provided by the International Consortium of Brain Mapping, and a diffeomorphic image registration algorithm (DARTEL) [22] was used for spatially normalizing tissue maps into stereotactic Montreal Neurological Institute (MNI) space. Finally, normalized gray matter maps (m0wrp1*), depicting the absolute amount of regional gray matter (GM) volume corrected for individual brain sizes, were smoothed with a standard 12 mm full-width-at-half-maximum (FWHM) [23] isotropic Gaussian kernel and used for further statistical analyses.

Group comparisons in local GM volume were calculated using a full factorial model comprising the factors: 1) level of moral reasoning (post-con vs. pre-con + con), and 2) high vs. low moral judgment competence. Although gender is not the focus of the present study, a considerable body of research has reported significant differences in GM volume of men and women. Therefore, we decided to include gender as a matter of interest as an additional factor in the model. Age was also included as a covariate of no interest. For a discussion of the necessary adjustment for gender and age in MRI studies, see [24].

Absolute gray matter thresholds of 0.25 were used to prevent edge effects located at the border regions of the tissue maps. To correct whole-brain results for multiple comparisons (p < 0.05, corrected), cluster extent correction procedures were used [25, 26]. Specifically, a cluster-defining height threshold (set at p<.001, uncorrected) was combined with an empirically determined extent threshold. The extent threshold is defined by the number of expected voxels per cluster. Importantly, cluster sizes vary with local roughness of the images and were thus adjusted depending on the smoothness of the data using non-stationary random field theory procedures [27]. In our study, the minimum cluster size was determined to be 243 adjacent voxels in all analyses.

## Results

### Group differences in personality traits

To analyze group differences in personality traits, we conducted a multivariate analysis of variance including the factors 1) level of moral reasoning based on the DIT-2 (post-con vs. pre-con + con), 2) high vs. low moral judgment competence, and 3) gender as independent variables. Dependent variables were the different test scores from the NEO PI-R (Neuroticism, Extraversion, Openness to Experience, Conscientiousness, and Agreeableness) and the test scores indicating self-esteem, sense of control, and self-efficacy. This analysis revealed higher scores in Openness to Experience (F(1,57) = 11.869, p = .001) and lower scores in Neuroticism (F(1,57) = 5.124, p = .027) for participants at the post-conventional level of moral development. We observed no differences in personality scales between moral competence groups (all ps>.145). Females differed from males by lower scores in the General Self Efficacy Scale (F(1,57) = 6.62, p = .013), the Rosenberg Self-Esteem Scale (F(1,57) = 5.254, p = .026), and the Sense of Control Scale (F(1,57) = 3.88, p = .054). More specifically, females were characterized by lower self-esteem and lower sense of control, including lower perceived control, mastery, and more perceived constraints, confirming previous reports on gender differences in these variables [28, 29]. Finally, moral judgment competence score (C score) and post-conventional reasoning score (N2 score) were slightly but not significantly correlated (r = .22, p = .076).

### Group differences in local gray matter volume

**Fig 1:**
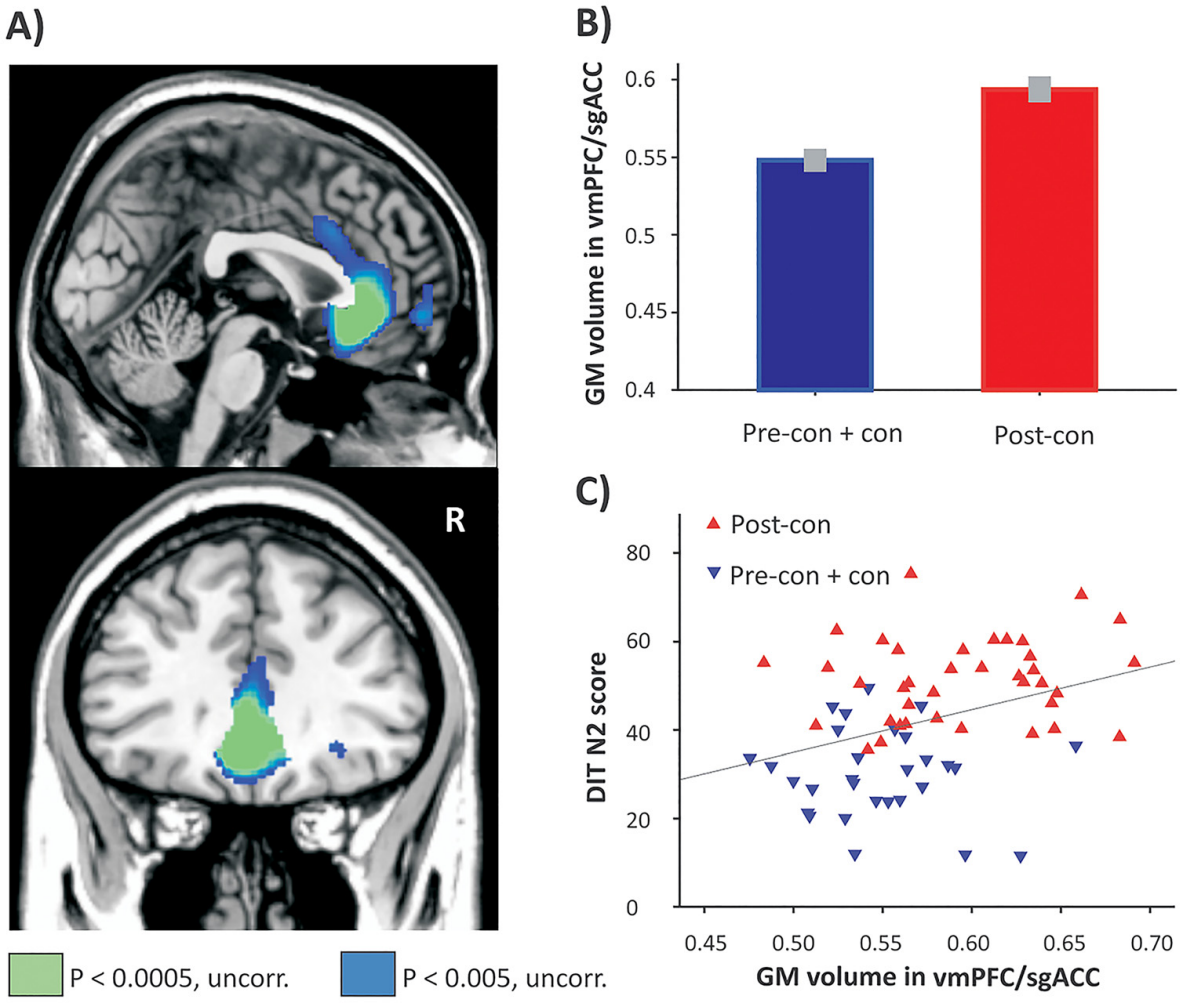
Moral reasoning and gray matter volume. Increased gray matter volume was found in the bilateral vmPFC/sgACC for participants who reached the post-conventional level (n = 38) of moral development compared to participants who did not reach that level (n = 29). For illustration purposes, gray matter (GM) volume was plotted in both groups in the region identified in the whole-brain analysis (B) and in relation with the degree of DIT-2 post-conventional reasoning (i.e., the N2 score) (C).

Participants who reached the post-conventional level of moral reasoning compared to those who have not (post-con>pre-con + con) showed significantly increased GM volume in the bilateral ventromedial prefrontal cortex (vmPFC) extending to the subgenual anterior cingulate cortex (sgACC; see Table 1 and Fig 1A and 1B). GM volume in the vmPFC/sgACC, the functional region of interest defined by the group contrast reported above, was associated with the degree of post-conventional thinking measured with the DIT-2 N2 score (r = .351, p = .004), as illustrated in Fig 1C. The correlation between vmPFC/sgACC volume and DIT-N2 score remains significant when controlling for the effect of moral judgment competence (i.e. the C score; partial correlation: r = .325, p = .008). GM volume in the vmPFC/sgACC, however, was also correlated with Neuroticism (r = -0.244, p = 0.050) and Openness to Experience (r = 0.31, p = 0.012). The reverse contrast (pre-con + con>post-con) revealed no significant differences. In this analysis, 38 subjects who reached the post-conventional level were compared with 29 subjects who did not reach that level. We did not find significant differences contrasting participants with high and low moral competence scores.

**Table 1:** Results from the whole-brain voxel-based analysis comparing local gray matter volume between the different groups split with regard to the level of moral reasoning.

Anatomical Region	Cluster size	Peak Z Scores	Peak MNI Coordinates		
			X	Y	Z
***Post-con* + *con* > *pre-con***					
Bilateral vmPFC/sgACC	2042	4.19	-9	26	-11
		3.44	9	28	-5
***Pre-con* + *con* > *post-con***					
No suprathreshold clusters					

Threshold was set as whole brain cluster corrected p < 0.05.

We also found significant gender differences. Women showed increased GM volume in a number of brain regions including the left inferior frontal gyrus, bilateral superior temporal gyri, bilateral hippocampi, left postcentral gyrus, left posterior cingulate cortex, as well as the left occipital lobe (see S1 Table). No significant differences were observed reflecting greater GM volume in men compared to women.

As reported above, GM volume in the vmPFC/sgACC, the functional region of interest defined by the group contrast post-con > pre-con + con, was also correlated with Neuroticism (r = -0.244, p = 0.050) and Openness to Experience (r = 0.31, p = 0.012). As reported in the Results section on group differences in personality traits, the two groups (post-con vs. pre-con+con) also differed in both dimensions (Openness: p = .001; Neuroticism p = .027). To assess the possible mediation of the differences in GM volume associated with the level of moral reasoning by both personality factors, we computed a supplementary and exploratory whole-brain model comprising the factors: level of moral reasoning (post-con vs. pre-con + con), high vs. low moral competence, gender, the covariates Openness to Experience and Neuroticism score, as well as age as a covariate of no interest. In this model, no differences were observed in GM volume associated with Openness to Experience. Subjects with higher scores in Neuroticism showed increased GM volume in the right posterior superior temporal gyrus. Moreover, in this analysis controlling for personality differences, subjects who reached the post-conventional level of moral reasoning compared to those who have not (post-con>pre-con + con) showed significantly increased GM volume in the dorsal ACC and the superior frontal gyrus (see Table 2). At a more lenient threshold (p = 0.005, uncorrected), we also found activation in the vmPFC/sgACC (peak MNI coordinates: x = -9, y = 26, z = -8; Z-score = 3.13; 430 voxels). In this model, no significant differences were observed contrasting participants with high and low moral competence scores. Results with regard to gender differences were similar to the results obtained in the model reported above.

**Table 2:** Results from the whole-brain voxel-based analysis comparing local gray matter volume between the different groups split with regard to the level of moral reasoning after controlling for personality differences.

Anatomical Region	Cluster size	Peak Z Scores	Peak MNI Coordinates		
			X	Y	Z
***Post-con* + *con* > *pre-con***					
R. superior frontal gyrus	294	3.78	14	68	1
R. dorsal ACC	363	3.50	5	15	31
***Pre-con* + *con* > *post-con***					
No suprathreshold clusters					

Note because the NEO data of two subjects were not available, this analysis compared 38 subjects who reached the post-conventional level with 27 subjects who did not reach that level. Threshold was set as whole brain cluster corrected p < 0.05.

## Discussion

In the present study, VBM was used to investigate how individual differences in moral reasoning and moral judgment competence are reflected in structural brain architecture. We compared GM volume of subjects with different moral reasoning styles and found that subjects characterized by a post-conventional schema preference, compared to those who have not reached that level of moral reasoning, showed increased GM volume in the bilateral vmPFC/sgACC. We also found that post-conventional moral reasoning was associated with increased Openness to Experience and decreased Neuroticism.

While Greene and Haidt [30] contend that no brain region is exclusively dedicated to moral reasoning, the finding of increased GM volume in the vmPFC/sgACC in subjects with a more sophisticated level of moral reasoning is supported by a body of functional neuroimaging studies demonstrating that moral decision making activates a neural network centered in the prefrontal cortex and in particular in the ventromedial PFC [4, 8, 31].

The vmPFC as well as the ACC are activated during a number of tasks requiring explicit moral judgments [5, 10, 32], the passive viewing of morally salient pictures (e.g., [6]), the monitoring of behavioral outcomes and adaptation of behavior in a manner that is ethically appropriate in a given social context [33], as well as the elicitation of charity, fairness, guilt, and other moral emotions [34]. VmPFC activity during moral reasoning has also been associated with social cognition [35, 36]. Young et al., for instance, found that vmPFC patients judged attempted (but not completed) harm as less blameworthy than accidental harm [37]. These results suggest that vmPFC patients may be unable to trigger an appropriate emotional response to information about harmful intentions, possibly supporting moral judgments based primarily on the outcome of an action [38, 39, 40]. The view that the vmPFC is a key structure in moral reasoning is further supported by Robertson et al., who demonstrated similar patterns of neural activity in the medial prefrontal cortex underlying sensitivity to moral issues of justice and care [41].

Differences in personality traits were also observed between subjects at different levels of moral reasoning. Subjects with a post-conventional schema preference were characterized by increased Openness to Experience and decreased Neuroticism compared to subjects at lower reasoning levels. To further assess the influence of both personality traits as a mediator variable on the structural findings, an additional covariate analysis was computed. This analysis, controlling for Openness to Experiences and Neuroticism, also revealed increased GM volume in the vmPFC/sgACC. In addition, we found increased GM volume for subjects at the post-conventional level compared to pre-conventional and conventional levels in the dorsal ACC and the frontal pole. The ACC in general has been associated with conflict processing and regulating both cognitive and emotional processes [42]. The dorsal ACC supports performing cognitive tasks, whereas the rostral or subgenual regions monitor new information that has potential affective or motivational consequences [43]. Hence, individual differences in moral reasoning might be consistent with affective and socio-cognitive developments. These developmental changes may comprise increased emotional stability, more elaborated cognitive processing based on abstract principles, or increased perspective taking and awareness of other people’s needs. Openness to Experience, furthermore, has been recently linked with leader efficacy in MBA students [44]. In other words, the developmental changes associated with post-conventional moral reasoning are reflected and mediated by differences in personality, which in turn are associated with changes in GM volume [45, 46].

A growing literature supports the notion of use-dependent brain plasticity, which holds that the size of a brain region is influenced by its use. This effect may be due to the fact that neurons that regularly “fire together, wire together” [47]. Increased synaptic connectivity and dendritic arborization may then lead to increased GM volume, which has been observed following cognitive and behavioral training interventions [48, 49]. Holzel et al., for instance, demonstrated changes in GM volume following an eight week mindfulness-based stress reduction intervention [50]. Further, in the moral domain, Lewis et al. used VBM and identified individual differences in moral values [11]. The authors found differences in brain regions supporting two moral sentiments: “individualizing,” which reflects harm, care, and fairness values, was associated with increased dorsomedial PFC volume; whereas “binding,” which reflects authority, loyalty, and sanctity, was related to bilateral subcallosal gyrus and left anterior insula volumes, respectively. The linkage of fairness with PFC volume fits well with our finding of increased GM volume in PFC in subjects at a post-conventional level of moral reasoning. However, while Lewis et al. investigated correlations between regional brain volume and specific moral values (e.g., fairness, authority, and care) across all subjects, our study focuses on contrasting GM volume between groups of subjects characterized by a specific pattern of shared moral values or (cognitive schema) preferences. In addition, our study is based on an established psychological model proposing a transition of individuals to a higher level of moral reasoning. Overall, Lewis’ study matches well our own aims to enhance knowledge of the neural basis of moral reasoning. However, as the studies differ in both theory and findings, further research is needed to enhance understanding of the relationship between the two studies.

Surprisingly, no brain structural differences were observed with regard to moral judgment competence. In a conceptually related study, Prehn et al. found that subjects with higher moral judgment competence showed decreased activation in the right dlPFC, vmPFC, and left posterior STS when making socio-normative compared with grammatical judgments [10]. Decreased activation in these brain regions has been associated with decreased cognitive control and lower moral judgment processing demands. Consistent with this view, moral judgment competence might represent an executive control function which is only reflected in functional—but not structural—brain architecture, in contrast to moral reasoning schema preferences which elicited significant structural changes at the group level.

The VBM analysis also revealed increased GM volume for women compared with men in a number of brain regions. Specifically, women were characterized by increased GM volume in the left inferior frontal gyrus, bilateral superior temporal gyri, bilateral hippocampi, left postcentral gyrus, left posterior cingulate cortex, as well as the left occipital lobe. These findings are consistent with a number of studies [51, 52, 53] showing that, relative to brain size, females exhibit greater cortical GM volume than males. Women, in particular, tend to have larger volumes in language-related brain regions [54], and also in frontal, parietal, and temporal cortices, ACC, and hippocampus [51]. Good et al. also demonstrated that men have greater volumes in the medial frontal cortex, amygdala, hypothalamus, paracingulate gyrus, and cerebellum [51]. These findings in men are not replicated by the current study.

It is presently unclear how different schema preferences measured with the DIT-2 map on to cognitive abilities that are central to moral reasoning, such as perspective taking, empathy, awareness of moral issues and the needs of other people. The broader role of empathy in moral reasoning is particularly intriguing and warrants future investigation, in addition to determining the relative contribution of empathic ability and social perspective taking to moral reasoning. As discussed above, the vmPFC and sgACC are activated during a number of socio-cognitive tasks (e.g., moral decision making, mentalizing, and conflict monitoring). For reviews see [30, 31, 35, 42]; thus the exact role of these brain regions during moral processing is also unclear. Therefore, future studies are needed to address how these different types of moral reasoning reflect different cognitive and affective abilities and to further elucidate the role of the vmPFC/sgACC during moral reasoning and development.

Furthermore, it must be noted that all study participants were MBA students who had many years of formal education, including a four year college degree. As discussed earlier, the relative homogeneity of study participants is considered a strength of the study. However, future work is needed to replicate these findings using broader and more heterogeneous samples. Future studies should also employ longitudinal designs to investigate changes in brain structure related to the development of moral reasoning.

The current study links individual differences in moral development with alterations in brain volume in a sample of MBA students. Post-conventional schema preferences were associated with increased GM volume in the bilateral vmPFC/sgACC. This executive brain region integrates social and emotional information central to moral cognition. When controlling for individual differences in personality traits such as Neuroticism and Openness to Experience, we also found increased GM volume in the vmPFC/sgACC as well as in other fronto-cortical regions including the dorsal ACC and the superior frontal gyrus. In sum, the current findings support an important role of cognitive and emotional processes in moral reasoning and provide initial evidence for brain structural alterations based on the stages of moral reasoning proposed by Kohlberg.

## Supporting Information

S1 Table Results from the whole-brain voxel-based analysis comparing local gray matter volume between female and male subjects. Threshold was set as whole brain cluster corrected p < 0.05.
(DOC)
