# Supplementary material for: Neural Correlates of Post-Conventional Moral Reasoning: A Voxel-Based Morphometry Study
Source: PLoS One. 2015 Jun 3;10(6):e0122914. doi: 10.1371/journal.pone.0122914 (PMC4454660; doi:10.1371/journal.pone.0122914)
Supplement: S1 Table — Threshold was set as whole brain cluster corrected p < 0.05. (DOC) [file pone.0122914.s001.doc]

Table S1. Results from the whole-brain voxel-based analysis comparing local gray matter volume between female and male subjects. Threshold was set as whole brain cluster corrected p < 0.05.

| **Anatomical Region** | **Cluster size** | **Peak Z Scores** | **Peak MNI Coordinates** | | |
| --- | --- | --- | --- | --- | --- |
|  |  |  | X | Y | Z |
| ***Females* > *males*** | | | | | |
| L. Posterior cingulate cortex | 679 | 4.42 | -2 | -36 | 49 |
| L. Occipital lobe | 733 | 4.18 | -24 | -84 | 12 |
|  |  | 3.92 | -26 | -76 | 25 |
| L. Superior temporal gyrus | 601 | 4.17 | -50 | -43 | 7 |
| R. Hippocampus | 755 | 4.07 | 22 | -36 | -8 |
| L. Parietal lobe | 1391 | 4.04 | -44 | -22 | 34 |
|  |  | 3.81 | -40 | -37 | 45 |
|  |  | 3.67 | -36 | -28 | 42 |
| L. Temporal lobe/hippocampus | 898 | 3.87 | -24 | -30 | -5 |
|  |  | 3.65 | -15 | -39 | 3 |
| R. Parahippocampus | 1010 | 3.84 | 54 | -28 | -2 |
| L. Inferior frontal gyrus | 363 | 3.65 | -38 | 8 | 30 |
|  |  | 3.53 | -45 | -1 | 24 |
| ***Males* > *females*** | | | | | |
| No suprathreshold clusters | | | | | |
